# Enacting intimacy and sociality at a distance in the COVID-19 crisis: the sociomaterialities of home-based communication technologies

**DOI:** 10.1177/1329878X20961568

**Published:** 2021-02

**Authors:** Ash Watson, Deborah Lupton, Mike Michael

**Affiliations:** University of New South Wales, Australia; University of Exeter, United Kingdom

**Keywords:** communication, COVID-19, digital media, digital technology, intimacy, sociality, sociomaterialism, the home

## Abstract

Significant restrictions on movement outside the home due to the global COVID-19 pandemic have intensified the importance of everyday digital technologies for communicating remotely with intimate others. In this article, we draw on findings from a home-based video ethnography project in Sydney to identify the ways that digital devices and software served to support and enhance intimacy and sociality in this period of crisis and isolation. Digital communication technologies had an increased presence in people’s domestic lives during lockdown. For many people, video calling software had become especially important, allowing them to achieve greater closeness and connection with their friends and family in enacting both everyday routines and special events. These findings surface the digital and non-digital materialities of sociality and intimacy, and the capacities opened by people’s improvisation with the affordances of home-based communication technologies at a time of extended physical isolation.

## Introduction

The unfolding crisis of the COVID-19 pandemic has drastically reshaped the day-to-day lives of many people around the world, including in Australia. Despite, at the time of writing, low national infection and death rates in Australia compared to many other high-income countries, measures introduced by government authorities to contain the spread of COVID-19 have had a major impact on Australians’ routines and relationships. The World Health Organization declared that COVID-19 was a pandemic on 11 March 2020. Australian federal and state governments began to announce international and national travel bans from this point onwards, and local movement restrictions soon followed. Social distancing rules and shutdowns of non-essential services began in Australia from 21 March, with this initial lockdown beginning to gradually ease from early June 2020 (Australian Bureau of Statistics, 2020a). Due to these restrictions, most Australians were unable to meet in person with others, beyond members of their households.

Given the currency and the dynamic nature of the crisis, the impact on Australians’ routines and relationships during this initial lockdown period has yet to be documented in detail. In this article, we offer an early contribution, reporting on some preliminary findings from a qualitative research project funded by the Australian Research Council which aims to identify how people live with and through their digital devices and the personal data these generate. ‘Living with Personal Data’, designed by Deborah Lupton and Mike Michael, was funded and commenced well before the COVID-19 crisis erupted, and therefore life during the pandemic was not the initial focus of this project. However, the reduced opportunities for in-person social contact have resulted in major changes to how our participants use and value everyday digital technologies in the home setting. Our project’s methods were also affected by the COVID-19 restrictions, and we were forced to adjust from in-person to ‘virtual’ ethnographic methods once these came into effect.

Drawing on the case studies from 12 virtual home visits we had completed between early April 2020 and the time of writing, in what follows we consider the diverse situated ways that digital media technologies were taken up by our participants to maintain connections with people outside their households during the period of pandemic-related home confinement. Adopting a sociomaterial theoretical perspective, we identify how our participants enacted intimacy and sociality with and through digital devices and software as they worked to maintain existing relationships. We document the human-digital-home assemblages of which our participants were part and consider the materialities of these practices by surfacing the relationality of bodies, spaces and objects. We are interested in both what our participants *say* about their digital technology use and what they *do*, and how these material-discursive elements have come together and are made to matter (Barad, 2003) in people’s strategies for coping with physical isolation during the COVID crisis.

We begin with providing a discussion of related research before detailing our research project. Our discussion of findings follows in three sections. We explore (1) the importance of visual elements in communicative technologies during this time, (2) the increased frequency of social contact in which our participants engaged and (3) the limitations of digital communication highlighted by our participants in their accounts. We conclude by discussing the significance of these practices in the context of the COVID-19 crisis, reflecting on the value of taking a sociomaterial approach to understanding digitised materialities of intimacy and sociality and outlining directions for future research.

## Background

A growing body of research adopts a sociomaterial perspective to examine how people come together with material objects to configure dynamic human-nonhuman assemblages that enact intimacy and sociality. The literature on the ‘materialities of care’ (Buse et al., 2018) works to surface the often unacknowledged spatialities, temporalities and materialities of formal and informal healthcare (Lupton, 2019). Similarly, discussions of ‘intimate entanglements’ (Latimer and López Gómez, 2019) and ‘mundane intimacies’ (Hjorth et al., 2018) draw attention to the taken-for-granted practices that bring humans and things together to generate forces, relational connections and agencies. These approaches emphasise that social relationships are dynamically configured with and through material objects. They focus on the everyday, affective and relational nature of enactments of materialities of intimacy and sociality.

The literature on digitally mediated intimacies and affects points to the significant opportunity for digital media to be used for expressions of affection, friendship, familial ties, emotional connection and concern for others. Sociomaterial research has found that paying close attention to the routine enactments of digital technologies can surface the complexities of these practices. For example, Pols’ (2012) work on ‘care at a distance’ in relation to devices used for telehealth notes that the common conceptualisation of communication using technologies as ‘cold’ and face-to-face interactions as ‘warm’ neglects the nuances of people’s experiences. Other studies have demonstrated how close relationships can be established or supported by digitally mediated interaction on social media platforms (Farci et al., 2017; Madianou and Miller, 2012) and locative media (Hjorth et al., 2018). In terms of family relationships, several researchers have shown that family members separated by long distances have found benefits in using digital media such as Skype and messaging apps (Longhurst, 2016; Madianou, 2016) for enacting transnational and intergenerational ‘ambient co-presence’ (Madianou, 2016).

Before the COVID physical distancing restrictions, many Australian households were already using mobile devices such as smartphones and tablet computers to coordinate with family members in the home as well as maintain connections with friends and family living further away (Baldassar, 2014, 2016; Baldassar and Wilding, 2014; Cabalquinto, 2017; Hjorth et al., 2015, 2018; Holloway et al., 2014; Zhao, 2019). For example, bringing together the literatures on the digitised home environment with that on transnational communication, Cabalquinto’s (2017) study of the digital practices of Filipino workers in Melbourne showed how the concept and space of ‘home’ were negotiated in and by these activities. He identifies the sociospatial and temporal dimensions of these practices of home, positioning the home as a dynamic assemblage of habituated practices conducted largely with and through the messaging and video conferencing apps used on the devices. His participants emphasised their preference for video calls over voice-only calls as a way of enacting ambient co-presence with their family members. Beyond talking, they used video calls to engage in playing instruments together, supervising homework or preparing and eating a meal together in real time, thereby performing family rituals that made their temporary dwelling feel more like home.

Researchers have begun to reflect on changes to the home environment and experiences of ‘being at home’ as part of initial COVID lockdown and quarantine restrictions in Australia. These commentaries have pointed to the increased burden placed on women to engage in care of family members during the initial stages of the pandemic (Craig, 2020; Nash and Churchill, 2020) and how physical distancing measures may have contributed to or exacerbated family violence (Strengers, 2020), difficulties in social connection and support for people with disabilities (Goggin and Ellis, 2020) and loneliness for older people who live alone (Neves and Sanders, 2020). The role of digital media in helping Australians connect with others and support mental well-being during periods of physical isolation has been alluded to in some of these pieces (e.g. Neves and Sanders, 2020).

An eight-stage longitudinal study of just over 1000 people conducted by the Australian Bureau of Statistics from the end of March (the ‘Household Impacts of COVID-19 Survey’) provides some initial quantitative insights into social and emotional changes wrought by COVID-related restrictions, including digital technology use for social connection. The first wave of the survey found that almost all respondents reported keeping their distance from other people and most were cancelling their plans to gather with friends and family (Australian Bureau of Statistics, 2020a). By the second wave, conducted in mid-April (Australian Bureau of Statistics, 2020b), almost half the respondents had experienced changes to their employment (including working fewer hours or losing their job), nearly one-third reported that their households finances had worsened due to COVID-19 and over 40% were struggling with feelings of anxiety. Just over half of the respondents said they had not had in-person contact with friends and family outside their household during the previous fortnight. However, nearly all reported contact using communication technologies, including verbal phone calls (92%), text or instant messaging (86%), video calls (67%) and email (42%). The third wave of the survey (Australian Bureau of Statistics, 2020c) found that most respondents were still keeping their distance from people outside their household and avoiding public spaces, but 22% reported feelings of loneliness. Three in five respondents also reported that their participation in screen-based activities, such as television and streaming services viewing, had increased.

To this developing picture of Australians’ experiences of the COVID crisis, our project’s findings contribute rich ethnographic material on people’s experiences with technology at home in lockdown, offering insights into how people enacted familial and other social relationships during the early months of the COVID-19 crisis.

## Details of the study

In the broader project on which this article draws, we investigate how Sydneysiders from a diverse range of backgrounds use digital technologies in and outside the home setting, and their understandings and practices related to the personal digital data generated from this use. There are two phases in this project: (1) fieldwork involving visits to people’s homes in different parts of Sydney (currently in progress), and (2) group workshops (yet to be conducted). In designing the home visit fieldwork, we were inspired by previous studies using video ethnographies to document Australians’ everyday experiences of using digital technologies in home settings. These studies have explored Australians’ household practices such as their use of broadband technologies (Kennedy et al., 2015), smart home devices (Strengers and Nicholls, 2018) and locative technologies (Hjorth et al., 2018).

For our home visits, we asked participants to move around their homes, showing us the digital devices they and their family or housemates used and explaining how they used the technologies. To facilitate further discussion about devices and data, participants were asked to draw maps, using pen and paper, of their digital devices in and outside the home in relation to each other, showing what personal data were generated by these devices and where these data travel. The final question asked participants to engage in a speculative exercise, imagining a new digital technology to best suit their needs.

At the time the COVID-related restrictions were announced, we had commenced the first phase of the project, but were forced to shift our ethnographic home visits to a completely digital method and implement the amended fieldwork protocol. Initial ethics approval was provided by the UNSW Sydney Human Ethics Research Committee, and a variation request to change the home visits to a virtual format in response to physical distancing restrictions was subsequently approved. This phase now involves either in-person (pre-COVID restrictions) or virtual (during COVID restrictions) home visits, lasting between 1 and 1.5 hours on average. All home visits discussed in this article were conducted in this modified, virtual way. Participants engaged in a video call with a member of the research team, video-recorded using the Zoom app, and used a mobile personal device (smartphone or tablet) to lead tours of their home and engage in the map-making activities.

During and following each virtual home visit, Watson composed ethnographic fieldnotes, creating a detailed case study for each participant that together with the fieldnotes included partial transcriptions and selected screenshots from the video recording of the home visit and photographs of the maps drawn by the participants. This corpus of research materials was used for our analysis. Working with our sociomaterial perspective, we reviewed each case study with the intention of surfacing the complexities of our participants’ enactments of digital devices. In addressing the question of how digital technologies were ‘made to matter’ (Barad, 2003) during this unprecedented time of physical separation from friends and family members, our analysis concentrated on identifying how our participants’ accounts and the visual materials gathered during the virtual home visits revealed how connection with others was enacted. We focused on the affordances of apps and platforms in and for the unfolding of sociality and intimacy at a distance.

Given the dramatic changes to Australia’s awareness of and response to COVID-19 from our first home visits in February to our virtual home visits in April, it is unsurprising that while the pandemic was not mentioned by participants in the early face-to-face fieldwork, COVID-related issues were raised by all participants in the virtual home visits without prompting by the researcher. As we detail below, discussions about the impact of the pandemic mostly concerned the significant impact of the lockdown on how much time participants spent in their homes and using their personal devices, and changes to how participants and their household members used these technologies for work, education, leisure and entertainment, and communicating and socialising with their close friends and family members compared with pre-COVID times.

It is on this last-mentioned use that our discussion in this article focuses, identifying the ways that digital devices and software served to support and enhance materialities of intimacy and sociality at a distance. We emphasise the embodied and sensory nature of how meaning and matter entangle, to better attend to the unfolding and generative ways that humans and nonhumans intermesh, and to critically consider what these assemblages can do and how they can ‘come to matter’ (Barad, 2003) in people’s everyday lives and social relationships during the COVID lockdown period.

### Participants

The home visit fieldwork phase as a whole will recruit a total of 30 participants. All participants are recruited from Sydney, with an equal number of women and men and a diverse range of ages, education and ethnic/cultural backgrounds (reflecting the cultural diversity that is characteristic of the Sydney population). To facilitate recruitment, a research company was commissioned to identify and screen potential participants from its volunteer research participant panels, using the sociodemographic characteristics identified above as sub-quotas. The virtual home visits completed thus far involve the following participants: nine women and three men, aged between 20 and 70 years, who were employed in a diverse range of fields and were either Australian-born or had lived in Australia for more than 5 years. We have given all participants pseudonyms, and to further protect their identity have generalised their self-descriptions of details such as suburb of residence, employment and ethnicity.

## Findings

### Overview

The participants had a diverse range of technological devices in their homes. All reported owning smartphones, a laptop and/or desktop computer, and using media streaming services. In addition, several people owned tablet computers, smartwatches, smart TVs or smart home assistants. All participants noted that due to the impacts of COVID-19 restrictions, they had recently increased the amount of time they were spending using these devices. Furthermore, they reported some changes in device use, including the style and frequency of their communications with family and friends using digital devices and software. Our participants spoke of a range of ways they were keeping in touch during the lockdown. Using text-based group messages, with multiple friends or multiple family members, was reported by nearly all of our participants. Many noted they also ‘still’ spoke to people on the phone. Due to the visual affordances offered by video calling, this was the most discussed and most affective medium for all of our participants. It is to detailing these technological practices, device affordances and their relational meaning for participants that we now turn.

### ‘Seeing people is more human’ – the importance of the visual

During the COVID-19 lockdown, digitised communication was used both for regular catch-ups that would usually take place by way of phone or video calls and for social encounters that would typically take place proximately. The convenience of being able to use video calling to engage in extended family and other group socialities is evident in Danielle’s account. Danielle, aged in her late 30s, lives in northern Sydney with her partner and two young children, works part-time in health services and is of Anglo-Celtic/European heritage. Danielle spoke about the central role that video calling has played in her familial relationships for several years now. She explained,

With the smartphone and the iPad, we do lots of video chatting. Our family lives in [another city], so we’re on video chat most days with the grandparents . . . We did even [video call] before the coronavirus, so we’ve been doing that for, well, since the kids were babies really. Being further away, we’ve sort of had to. (Video interview)

In Danielle’s experiences, the affordances of the multisensory features of video calling technologies were meaningful for her and her children’s relationships with family in particular:

I guess it’s different from a phone call because you feel like you’re actually sitting, you know, getting a visual on someone, you feel like you are actually talking face-to-face. I like that aspect of it, especially for my parents getting to see the kids. Because otherwise they grow really quickly and if you don’t actually get to really see them, you know, they change quickly, so that’s been nice. (Video interview)

While video calling the grandparents was a regular practice in Danielle’s household, other forms of sociality had moved to mediated communication only since the COVID-19 lockdown. Danielle said that she had been video calling with an established group of friends in lieu of their usual in-person brunch catch-ups. She commented that despite this new mode of catching up, her sense of ‘being with’ the group in the usual way remains:

With friends, you know, you can sit there and have a glass of wine and we get our cheese platters ready. And it feels like we’re sitting, you know, we could be in the middle of a restaurant, the four of us chatting, nothing’s really changed. So it is nice having that visual to go on as well. (Video interview)

For Danielle, the affective texture of her digital socialising is enhanced with the addition of food and wine so that the friends can see each other eating and drinking as well as chatting. The social connection afforded by a video calling platform was also something Danielle was happy her children were able to experience with their friends during COVID times. She emphasised how the affordances of mobile devices to readily accompany her children’s movements around the house helped them to connect with their friends:

A lot of my friends with kids the same age, who my kids are friends with, because they’re missing out seeing their friends, they’ve been using [video calling] with their friends as well. Little virtual playgroup dates [laughs] . . . It’s a bit chaotic, they usually just run around with the phone, you know, spinning in circles and yelling at each other. (Video interview)

The fieldnotes from the home visit with Dev also highlight the affordance of connection that video calling seemed to offer better than voice-only communication. As an immigrant to Australia from another country, many of Dev’s family members are overseas. Dev, aged in his late 60s, works part-time from home in professional services. He lives with his wife in southern Sydney, has an adult daughter and is of South Asian heritage. Like Danielle, Dev has used digital communication technologies to maintain familial relationships for a long time, but has more recently moved to video calling as his preferred mode:

First off, we talk a bit about the apps Dev uses most. He uses his smartphone a lot, mostly to communicate with friends and family members . . . He uses apps like WeChat and WhatsApp a lot, to stay in touch with family and friends in Australia and across the world, preferring direct individual and group messages rather than social media platforms to stay in touch with people. Dev says he is now video calling with his friends on the platforms they already use rather than just voice calling. I ask him why he thinks this is and he replies that seeing people’s faces is ‘more social’ and ‘more human’ – it helps them better connect and be together in the current crisis. (Fieldnotes)

This intimate ‘more human’ ambient co-presence was something Holly sought throughout the lockdown. Holly, aged in her late 20s, lives in central Sydney with a housemate, works full-time in real estate and is of Anglo-Celtic/European heritage. In response to the speculative question about an ideal digital technology she would like to see invented, Holly said she wished for a hologram device:

I could see my friends more and, you know, just like catch up with family more so . . . my best friend lives in [a different Australian city], and if I could hologram her to be closer to me sometimes that would be awesome. That’s probably it, more like connectivity and wanting to be closer to people. But maybe that’s also just coming out more at the moment because we feel so disconnected from people, maybe that’s why I’m feeling like that would be such a good one for me right now. (Video interview)

Tracy is in her early 40s and works part-time in customer service. She lives in southern Sydney with her husband, has no dependents, but was helping with her niece’s home schooling during the lockdown, and is of Southern European heritage. The conversation with Tracy was particularly illustrative of the value of the importance of multisensory and affective engagement during the COVID period, particularly as she was anxious about the health and well-being of her sister and brother-in-law, who were working in essential services:

You worry for them, you’re like, oh I hope you’re OK. Like, if I see them face-to-face, I will know from their reaction or whatever – it’s getting me emotional even thinking about this – but you get an idea, oh are they OK, are they coping? . . . I just – I don’t know, [video calling] is good as in you can see someone and, you know, immediately you can see how they are, ‘cause if you ring them at 9 o’clock in the morning you can see what they look like. And you’re like, OK, she’s brushed her hair today and, you know [laughs]. (Video interview)

Across these case studies, participants see additional visual elements of digital devices affording a co-presence with close relations that is more social and more intimate than, for instance, text messaging or voice calling. Something that became clear for each of our participants was the significance not only of ‘getting a visual’ on someone, to use Danielle’s words, but what Tracy speaks to when she talks about preferring video messages over more ‘flat’ or unimodal forms of communication.

### ‘You kind of make the effort a bit more now’ – increased frequency of contact

The case studies also show that the frequency of digitised communication had increased during the crisis. Like Danielle, Dev had previously used digitised communication more frequently over the years to keep in contact with his family, but this had increased significantly since the COVID-19 crisis had erupted:

I ask about the changes in technology over the past five years and he tells me it’s considerable – he used to have to go to the post office, for instance, to call his family in [South East Asia], and it would cost $6 for 3 minutes, whereas now he does it from his smartphone and it’s ‘free’ (other than the internet data cost, he notes) . . . I ask how his technology use has changed in recent times, and Dev reflects that it hasn’t changed in terms of what he’s using, but he feels like he’s using technology a lot more day to day. He’s chatting with friends more, for example. (Fieldnotes)

This was also the case for Lucas, who works full-time in project management, is aged in his early 40s and lives in Sydney’s west with his wife and young child. Lucas is of Anglo-Celtic/European heritage, with a history of recent immigration in his family. He explained having multiple group chats for family, friends and colleagues who live around the world:

Lucas really valued being able to maintain ongoing relationships with friends and family overseas, and keep up to date with what’s happening ‘back home’, by having ‘everyday’ contact – so he can still be actively part of his extended network/familial collective from a distance. (Fieldnotes)

Chris similarly noted that his use of digital technologies for communicating with friends and family had changed during the lockdown period, not so much in terms of the devices or software he was using, but rather in frequency. In his late 20s, he lives in inner Sydney with his partner and a housemate, currently works full-time from home in education/training and is of Anglo-Celtic/European heritage. Chris characterised this change as involving ‘making more of an effort’ to contact friends and family:

We’re doing the occasional Zoom call or kind of, you know, FaceTime call. And I mean, we did that all the time anyway, before. I guess all my friends knew how to use that as well, so it’s not new. But you kind of make the effort a little bit more now, because it’s kind of the thing to do and you’re not seeing anyone [in person]. (Video interview)

This additional effort to connect was also evident for Kim – a woman in her mid-50s who works full-time in education, lives in Western Sydney with her husband and two adult children, and is of Anglo-Celtic/European heritage. While usually ‘everyone just watches their own thing in their own rooms’, both within their household and with friends further afield, Kim’s family were spending more time together:

We’ll have a Zoom meeting with other friends and we play games . . . It’s a new lockdown thing, every maybe three weeks we play Kahoot with a group of adults that are all over Australia, friends of ours . . . That’s probably once every three weeks on a Friday night with a drink and some nibblies . . . We wouldn’t normally see those people because they live interstate, so because they’ve been locked down as well they’re going ‘Hey, how about we just all get together and play Trivia and talk?’. (Video interview)

Similar experiences were shared by Sue, who is aged in her early 50s and lives with her husband and two children in eastern Sydney. Sue works part-time in professional services, currently from home, and is of Anglo-Celtic/European heritage. She said that she was video calling more with her elderly parents who they recently have been unable to see in person, saying this had ‘been good for the kids’. She also noted making more of an effort than usual to connect with friends:

We did have a Zoom chat with some friends who live in Spain, and their kids, which was something we’d never have done before. So that was really good to do that by the phone, by the smartphone, and I have had a chat or two with some friends who are scattered around the world and some who are in Sydney as well. (Video interview)

While most people’s accounts focused on using digitised communication to maintain and increase the frequency of regular catch-ups with friends and family during the COVID lockdown, there were some examples of special events. Some meaningful examples for Melissa were her experience attending a virtual 40th birthday party and an online wedding. Melissa is aged in her early 40s, lives in inner Sydney with her partner, has no dependents, works in the cultural sector and is of Anglo-Celtic/European heritage. The ethnographic fieldnotes detail the following:

Melissa really warmly recounted her experience attending a virtual wedding recently. She and her partner did more than ‘watch’ this, which they could simply have done – instead, they got dressed up, ordered take-away to be delivered at the right time to have dinner, as if they were eating something special at the reception. After the ceremony, the specialised virtual platform which the bride and groom used to host the event meant that Melissa and her partner were also able to hang out in ‘breakout rooms’, where they could chat with a small group of friends as if they were sitting at a table together at the wedding reception. She noted that there was a ‘chat routlette’ style option too, where you could be paired with a random person on the virtual guest list, and would have six minutes to talk with each other before being randomly allocated to another conversation – she wasn’t really up for this, she laughed, but liked that this was an option. (Fieldnotes)

Reflecting across the varied novel and more typical communication-events in which she had recently participated, including the birthday party and wedding, Melissa said,

This time has definitely made me connect with people more than I might have otherwise, and people [who are] remote as well. Yeah. And you know that people are home, so you can schedule a chat, you know, they can’t go anywhere [laughs]. So it’s like, let’s hang out, what else are you going to be doing? I actually do have three things scheduled for Saturday night, I don’t know how that happened! (Video interview)

Another notable example, on which both Melissa and Tracy reflected, was the Easter period as a catalyst for their family’s adoption of more group-based conversations, especially via video calls or messages. In Tracy’s case, the fieldnotes capture how

Easter for her family was the real point of learning and change. It was an important time of the year where they’d usually all get together, and it showed that she and her siblings and parents can and need to stay in touch through this time via apps and platforms like Marco Polo – a messaging service she prefers, because it’s easy to send short video messages of themselves back and forth rather than texts or only voice messages. (Fieldnotes)

Melissa’s and Tracy’s example of the transformation of significant cultural rituals and celebrations to online engagements demonstrates how the COVID lockdown crisis forced people to improvise with new ways of enacting these kinds of socialities and intimacies.

### ‘It’s not real’ – the limitations of digitised communication

While Melissa appreciated the opportunity to digitally engage in special events and everyday interactions with friends and family, she also spoke about the differences or limitations with what these devices and software ‘capture’ of in-person experiences:

It’s a different experience. Being part of a musical or going to a gig, or anything like that, is just being around people and having a drink and having a chat around it as well, so I think that [the digital event] doesn’t quite capture it. (Video interview)

On the limitations or challenges of these digital media, she also noted,

I find [video calling platforms] hard if it’s a really big group chat, I find it hard to have any rapport, it’s difficult . . . the kind of technology means it’s more challenging than in real life. That’s the biggest con for me . . . If you’re in a group situation at a venue or at a pub or whatever, just having a chat, it makes much more sense. (Video interview)

Melissa also spoke about how the increased time spent using devices was not without its drawbacks. As the fieldnotes reflect,

Melissa definitely seemed a bit exhausted by the time she was spending using technology at the moment – her ‘screen time’ had really increased, and working on a computer all day plus being as digitally social as she has been seems quite taxing physically and emotionally. She spoke about being tired of looking at her screen so much and of the interpersonal/social difficulties of having group conversations over Zoom, and connected these possibly discrete feelings where her very embodied way of being and the affordances (and limitations) of the device meet. (Fieldnotes)

In her own words, Melissa said,

It’s just that whole thing of, that you’re looking at a screen all day, if you’re working on a screen or, you know, it’s just constant and gets a little bit tedious. You don’t want to be – you can’t dissociate the device that you’re working on from the one you’re using for social interaction . . . Someone I was working with the other day, he was like, ‘I’m just picking up the phone to call you because I don’t want to video chat again’. (Video interview)

Sue made some similar observations, noting that the stressors of the COVID pandemic shaped both what kinds of connections she sought out and how she felt about them:

With friends [who she’d usually see in person], we’ve been a little bit like hermits to be honest, over the last couple of months. And it’s been really hard trying to work from home, my husband’s working from home, both kids have been home schooling . . . So we haven’t really, like we have just kind of been a bit tired and a pretty depressed about the whole thing [laughs]. Watching the news and watching Netflix and trying to get outside a bit and that’s been pretty much it. (Video interview)

For Tracy too, while she commented that she was appreciative of the opportunities to see as well as hear her relatives using video calling, she was still reluctant to characterise these kinds of encounters as ‘real’ compared with face-to-face interactions:

I think, yeah [video calling] is good because it keeps you in touch with people. But there’s nothing like being face to face with people. Like, you know, you don’t get if somebody’s OK, you know, *really* OK . . . And, yeah, I don’t know, like, I just, I feel like it’s good because at the moment, currently, we can see people, but I don’t think – it’s not normal, like, it’s artificial, you know what I mean. Well, it’s not really artificial, because you’re seeing the person, but it’s not *real*. (Video interview)

As these comments suggest, people can ‘get together’ through the affordances of digitised communication technologies. However, while digital communication has become normalised as part of everyday life for all of our participants, there are many sensory and embodied dimensions of such interactions that typically never feel as ‘normal’ or as ‘real’ as proximate encounters.

## Discussion and conclusion

Our fieldwork has identified some of the ways in which the demands of restrictions on proximate encounters with other people have resulted in both an expansion and an intensification of intimacy and sociality at a distance using digital communication technologies. The COVID-19 crisis disrupted everyday routines and practices of sociality for many people. These have shaped their digital media practices, the affective labours of their kin-work (see Di Leonardo, 1987) and their experiences of the pandemic *as a crisis* (see, for example, Baldassar, 2007, 2014). Mundane enactments of digitised forms of communication changed in response to people’s need for connection and maintaining relationships. In other words, there was a reconfiguration of what above we called ‘human-digital-home assemblages’ in which our participants made changes to their use of familiar apps and platforms while confined to their homes.

The generative ways that the affordances of these reconfigured assemblages opened opportunities for sociality in the pandemic context reveal how digital devices came to matter for people in their close relationships. The need and desire for remote communication that felt connected and personal were central in how all of our participants use their digital devices and relate to why they feel they spend significant amounts of time using personal technologies. Our participants reported an increase in the frequency of technology use and the time they spent communicating with close friends and family. Here, we might say that intimacy and sociality at a distance were partly enacted through the frequency of contact. This was not only a matter of ‘checking up’ on the well-being of friends and relatives deemed more or less vulnerable, but of a sort of extended conversation or exchange that is more like ‘checking in’.

We note that these increases were not uniform for people or are uniformly sustained across their relationships. There were variations concerning with whom our participants nurtured novel digital connections. Melissa, for example, stopped using video calling with colleagues, and Sue had not substituted her face-to-face socialising with friends who she would usually regularly see with any digital alternatives during lockdown. As Madianou and Miller’s (2012) work highlights, the quality of existing relationships impacts and is itself augmented by different communicative modes. For our participants, their discussions about the kinds of people they did make the effort to connect with digitally were more strongly redolent of an affective and relational significance. In these relationships, video calling was the most significant medium, allowing participants to achieve greater closeness and connection with their friends and family member as part of both everyday routines and special events. These people included those with whom they were already close, such as parents or children, and those about whom they were more concerned in relation to the COVID crisis, such as siblings working in essential services or overseas friends.

In many ways, the findings from our research in the time of COVID-19 lockdown echo those of pre-COVID-19 studies in Australia on home-based communication technologies for connecting with friends and family based in remote locations (Baldassar, 2016; Cabalquinto, 2017; Holloway et al., 2014; Zhao, 2019). However, there were some novel elements that reflect the sudden and dramatic change in people’s social and family lives when their movements were largely limited to short excursions outside their homes. We found that social norms around mediated communication and intimacy changed in response to the lockdown restrictions. The impact of physical distancing meant that for most of our participants, these digital communication technologies took on much greater importance than in pre-pandemic times as a way of breaching distances that in some cases may have been only streets or a suburb or two away. Major events and celebrations that would never have been conducted virtually suddenly were celebrated online. People who may never have needed to rely on video calling found themselves using it to a far greater extent to maintain their social and familial relationships. In this respect, participants’ human-digital-home assemblages were reconfigured in ways that expanded their enactment of ‘home’. As such, rather than using video technologies to make them feel more ‘at home’, these devices and media were employed in a way that helped people who were confined to their homes to escape this sense of confinement and isolation.

For our participants, the devices and software that were incorporated into their human-digital-home assemblages generated feelings of connection and co-presence that offered them comfort. In this sense, the kind of ‘warmth’ and ‘closeness’ that people can feel in response to healthcare that is mediated by digital technologies (Pols, 2012) was clearly evident. Particularly in relation to the participants’ reflections about why they value video calling and increased frequency of contact, we see that it is a layering of sensory affordances that helps to generate the affective forces of intimacy, closeness and care for others. This is not to suggest that the more sensory elements a device affords, the more personal a conversation using that device will feel. The same affordances that are valued in small group conversations can, in large group situations, create a distance rather than intimacy and a tiring sense of tedium rather than – and as well as – care and connection.

The complexities and relational pressures of sustaining relationships via digital technologies, as Cabalquinto (2018: 259) argues, can lead to a ‘paradox of intimate connectivity’. The intra-action of emotional labour and technological parameters means long-distance relationships are sustained with and through intimacy and care, as well as sadness, obligation and coping tactics such as the negotiation of visibility (see also Nedelcu and Wyss, 2016). However, a particular assemblage of familial/social relations and sensory engagements, compared to other modes of communication and in lieu of physical proximity, does give rise to affective qualities many people want from their conversations with friends and family – an ‘intimate co-presence’ (Hjorth et al., 2015), digitally bridged across distance and possibly sustained over time. These include combinations of real-time sight plus sound, hearing someone’s voice while seeing their face, sitting at a dining table together, dressing in appropriate clothing for the occasion, playing boardgames together, consuming food, having a drink or moving in space together. Such practices, because they are digitally mediated via a video platform, can be seen as both the ‘doing’ and ‘displaying’ of family (Finch, 2007), significant even when ‘within family’ rather than for an external audience (Finch, 2011). In these accounts, the digital and more-than-digital materialities of intimacy and sociality come to the fore.

On the more-than-digital, social and structural living conditions are significant in that people can engage digital media to sustain sociality and intimacy. As Piele’s (2018: 135–136) research highlights, personal differences such as technological familiarity *and* structural differences such as costs resulting from telecom service providers and gendered expectations of kin-work responsibilities condition ‘possibilities of communication’ within families. Socioeconomic contexts (see Peile, 2018: 142–143) and relational tensions are often intensified by distance and crises (and vice versa) where forms of connection, care and support are required that are beyond the ‘routine’ or ‘ritual’ (Baldassar, 2014; Baldassar and Wilding, 2014). These are especially pressing considerations as the COVID crisis unfolds and continues to impose degrees of physical and social distance. Work that attends to material societal conditions and the affective forces of social imaginaries on the future technologies of relationships, considering the indeterminate end of COVID life, will contribute significant insight to this literature.

There are a number of additional aspects of the human-digital-home assemblage during COVID lockdown that would also benefit from further investigation. For example, how the space of the home (e.g. the backdrop to video calling encounters), modes of self-presentation (e.g. how an individual dresses, the food and drink they consume during digitised encounters) and digital functionalities (e.g. deployment of the blurring facility) are used can affect the ways in which intimacy and sociality at a distance come to be enacted. Moreover, the human-digital-home assemblage is of course entangled with other assemblages which potentially play a role. These include, for instance, governmental announcements about the projected length of lockdown, the slowing of bandwidth by providers, working or education at home contingencies. Put simply, in exploring how the material and sensory qualities of digitally mediated communications help make those communications matter, we also need to situate those qualities within expanded assemblages that do justice to the sociomaterial complexities that participants daily face. Future fieldwork and analysis of our case studies will be devoted towards some of these questions.

What our present findings do highlight is the importance of digital communicative technologies in times of crisis and distance. This novel social and structural situation of distance brought about by the COVID pandemic reveals some of the digital media practices and meanings significant for how people affectively manage and relationally respond to this distance. Layered sensory affordances and possibilities for ongoing and increased frequency of contact – where people can see *and* hear each other, and collectively ‘check in’ rather than be ‘checked up on’ – can work to materially heighten the intimacies and socialities which sustain these relations. Importantly, for our participants, these changes are not seriously seen or desired as a ‘new normal’ in an ongoing way. They are a contingent supplement born of necessity, different from and less ‘real’ or ‘human’ than proximate relationships, but nevertheless of central significance and meaningful in the crisis context.
